# Hierarchy of Dysfunction Related to Dressing Performance in Stroke Patients: A Path Analysis Study

**DOI:** 10.1371/journal.pone.0151162

**Published:** 2016-03-08

**Authors:** Takaaki Fujita, Hirofumi Nagayama, Atsushi Sato, Yuichi Yamamoto, Kazuhiro Yamane, Koji Otsuki, Kenji Tsuchiya, Fusae Tozato

**Affiliations:** 1 Department of Rehabilitation, Faculty of Health Sciences, Tohoku Fukushi University, Sendai, Miyagi, Japan; 2 Department of Rehabilitation Sciences, Gunma University Graduate School of Health Sciences, Maebashi, Gunma, Japan; 3 Department of Rehabilitation, Faculty of Health and Welfare, Kanagawa University of Human Services, Yokosuka, Kanagawa, Japan; 4 Department of Physical Therapy, Yachiyo Rehabilitation College, Yachiyo, Chiba, Japan; 5 Department of Rehabilitation, Northern Fukushima Medical Center, Date, Fukushima, Japan; 6 Department of Rehabilitation, Japan Community Healthcare Organization Gunma Chuo Hospital, Maebashi, Gunma, Japan; Fraunhofer Research Institution of Marine Biotechnology, GERMANY

## Abstract

Previous reports indicated that various dysfunctions caused by stroke affect the level of independence in dressing. These dysfunctions can be hierarchical, and these effects on dressing performance can be complicated in stroke patients. However, there are no published reports focusing on the hierarchical structure of the relationships between the activities of daily living and balance function, motor and sensory functions of the affected lower limb, strength of the abdominal muscles and knee extension on the unaffected side, and visuospatial deficits. The purpose of this study was to elucidate the hierarchical and causal relationships between dressing performance and these dysfunctions in stroke patients. This retrospective study included 104 first-time stroke patients. The causal relationship between the dressing performance and age, time post stroke, balance function, motor and sensory functions of the affected lower limb, strength of the abdominal muscles and knee extension on the unaffected side, and visuospatial deficits were examined using path analysis. A hypothetical path model was created based on previous studies, and the goodness of fit between the data and model were verified. A modified path model was created that achieved an almost perfect fit to the data. Balance function and abdominal muscle strength have direct effects on dressing performance, with standardized direct effect estimates of 0.78 and 0.15, respectively. Age, motor and sensory functions of the affected lower limb, and strength of abdominal muscle and knee extension on the unaffected side have indirect effects on dressing by influencing balance function. Our results suggest that dressing performance depends strongly on balance function, and it is mainly influenced by the motor function of the affected lower limb.

## Introduction

Various physical and perceptual dysfunctions such as motor paralysis, sensory disturbance, muscle weakness, and visuospatial deficits can negatively impact an individual’s independence in the activities of daily living (ADL). A stroke can cause multiple dysfunctions simultaneously, resulting in the need for care and substantial assistance. Understanding the causal relationships between various dysfunctions and ADL is important to facilitate effective stroke patient rehabilitation. However, the relationships between specific deficits and ADL are not simple. For example, it has been reported that sensory disturbances in stroke patients are associated with independence in ADL [1], mobility [1], motor functions of the upper limb [2], and balance function [1]. On the other hand, other studies have reported that balance functions in stroke patients are associated with independence in ADL [3] and the motor functions of the affected upper limb [4]. When integrating this information, we presumed that sensory disturbances deteriorate balance functions, which consequently reduces the independence in ADL and the motor function of the affected upper limb. This reasoning suggests that the various dysfunctions caused by a stroke are hierarchical, and these dysfunctions induce complex effects on ADL. However, to the best of our knowledge, there are no published reports that investigate the hierarchical relationships between various dysfunctions and ADL.

Dressing is an ADL that is difficult for stroke patients to perform [5]. Previous studies have reported that a stroke patient’s level of independence in dressing themselves is associated with balance function [6,7], motor and sensory functions of the affected limbs [8], abdominal muscle strength [9], and visuospatial deficits [10]. As stated above, there is a possibility that these dysfunctions affect the level of independence in dressing not in parallel, but in a hierarchical manner. However, the hierarchical structure of the relationships between the level of independence in dressing and various dysfunctions has not been clarified. The findings on which dysfunction is directly or indirectly related to the level of independence in dressing and those on the degree to which each dysfunction affects the level of independence in dressing will be very useful for planning and executing rehabilitation strategies. Therefore, the purpose of this study was to elucidate the hierarchical structure and causal relationships between the level of independence in dressing and balance function, motor and sensory functions of the affected lower limb, strength of the abdominal muscles and knee extension on the unaffected side, and visuospatial deficits using path analysis.

## Materials and Methods

### Subjects

This study included 104 patients with stroke who participated in a rehabilitation program and were discharged from a hospital convalescent rehabilitation ward from April 2011 to February 2014 (Table 1). The study inclusion criteria were: diagnosis of a first onset cerebral hemorrhage or cerebral infarction; diagnosis of a unilateral supratentorial hemispheric lesion; being at least 4 weeks from stroke onset; and having at least a 2-week stay in the hospital convalescent rehabilitation ward. Exclusion criteria of subarachnoid hemorrhage and missing records were applied to all assessments mentioned below (Procedures and Data Collection). All patients participated in a conventional in-hospital stroke rehabilitation program that included occupational therapy, physical therapy and speech therapy. The study protocol was reviewed and approved by the institutional ethics review board of Northern Fukushima Medical Center (No.56) and Tohoku Fukushi University (RS141201). Participant informed consent was not required because of the non-interventional, retrospective study design.

**Table 1:** Demographic, Stroke-related Characteristics of Study Subjects^a^.

	Mean ± SD
Age, y	71.2 ± 13.4
Time post stroke, day	95.8 ± 37.5
Males, %	59.6
Right sided hemiplegia, %	46.2
FIM^®^ Dressing item (1–7)	4.3 ± 1.8
Berg balance function scale (0–56)	35.9 ± 18.5
Affected L/E motor function of SIAS (0–15)	10.2 ± 5.1
Affected L/E sensory function of SIAS (0–6)	4.3 ± 1.8
Abdominal muscle strength of SIAS (0–3)	2.1 ± 1.0
Unaffected knee extension strength of SIAS (0–3)	2.6 ± 0.7
Visuospatial deficit of SIAS (0–3)	2.7 ± 0.8

Abbreviations: SIAS, Stroke impairment assessment set; L/L, Lower Limb.

^a^ N = 104

### Procedures and Data Collection

This was a retrospective study by design that analyzed data from electronic medical records from the time of discharge. The variables used in this study were selected on the basis of previous reports (referenced in the Statistical Analysis section) where these variables were studied and associated with dressing performance or balance function. The FIM^®^ instrument [11] subscores for dressing the upper and lower body were used to assess each participant’s level of independence in dressing. The FIM^®^ instrument assesses the amount of assistance required in ADL and is rated on a 7-point scale, with a higher score indicating better performance. The FIM^®^ instrument’s scoring criteria were as follows: a score of 7 indicates complete independence, a score of 5 indicates supervision or setup, a score of 3 indicates moderate assistance, and a score of 1 indicates total assistance [11]. The reliability of the FIM^®^ instrument has been confirmed in stroke patients [12,13]. In this study, the lower score on FIM^®^ instrument for dressing the upper and lower body was regarded as the level of independence in dressing. The Berg Balance Scale (BBS) [14] was used to assess participant balance function. BBS is designed to measure balance among the elderly. It assesses an individual’s static and dynamic balancing abilities through the performance of functional tasks, including balance while sitting and while standing on 1 foot. It is composed of 14 items scored on a 5-point scale ranging from 0 to 4 points. The maximum total score is 56 points, with a higher score indicating better balance function. A BBS score of 40 points was reported as a cut-off value for independent functional walking ability among stroke inpatients [15], whereas achieving a BBS score of 44 points was the reported requirement for independent dressing [6]. The reliability and validity of this scale have been confirmed in stroke patients [16]. The Stroke Impairment Assessment Set (SIAS) [17] was used to assess motor and sensory functions of the affected lower limb, strength of the abdominal muscles and knee extension on the unaffected side, and visuospatial deficits. SIAS is used to comprehensively assess impaired functions due to stroke. Each item is rated on a 3- or 5-point scale, with a higher score indicating better function (S1 Table). These tests also have confirmed reliability and validity [18,19].

### Statistical Analysis

In this study, the total score for the motor function tests for the affected lower limb on SIAS (the hip flexion test, knee extension test, and foot-pat test) was used to assess the motor function of the affected lower limb; while the total score for the sensory function tests for the affected lower limb on SIAS (light touch and position test) was used to assess the sensory function of the affected lower limb.

**Fig 1:**
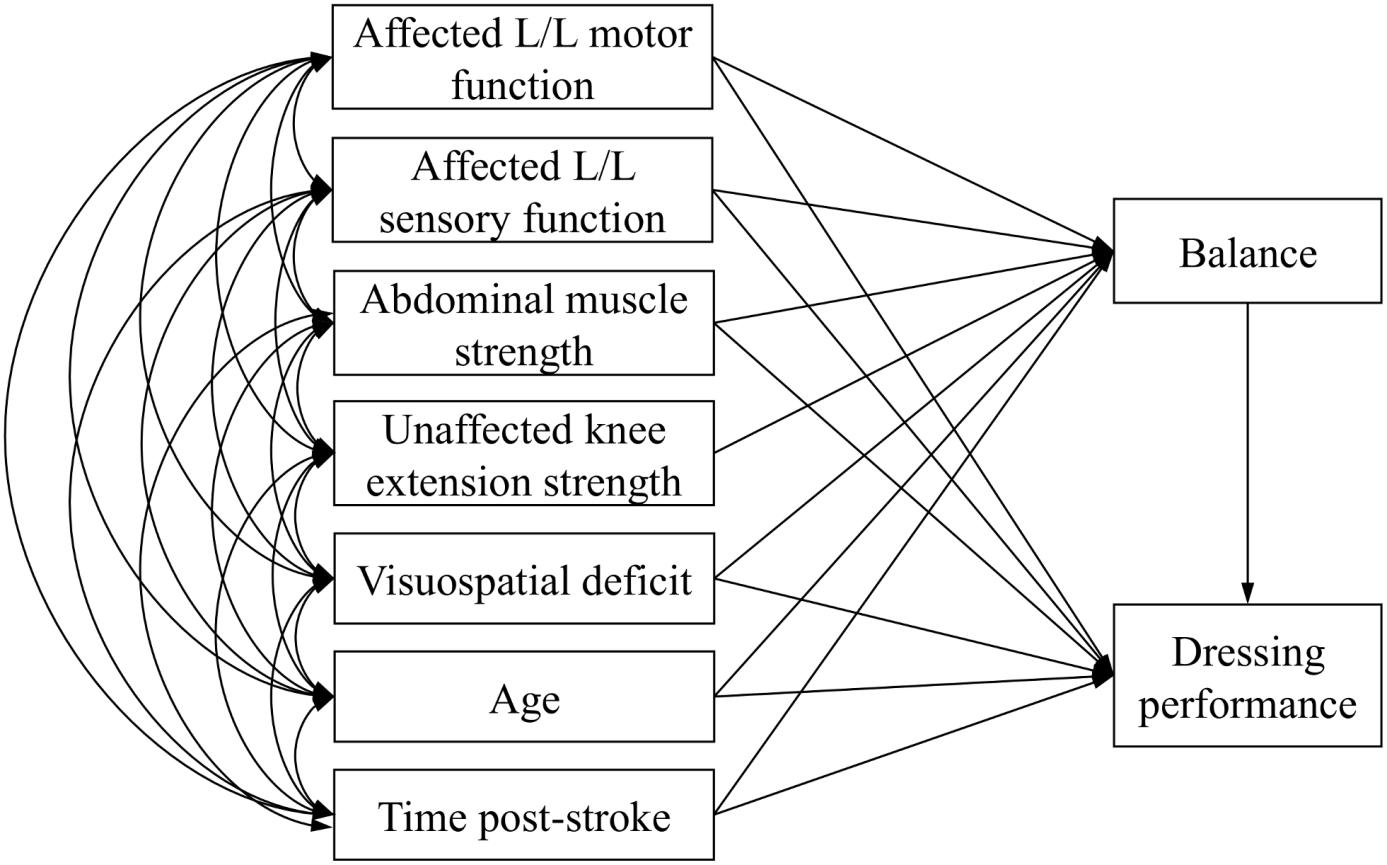
Path diagram of the relationship between the level of independence in dressing and physical and visuospatial dysfunctions. Abbreviations: L/L, Lower limb.

Path analysis based on structural equation modeling was performed to clarify the hierarchical structure of the relation and causal association between the level of independence in dressing and various dysfunctions. A hypothetical path model was created on the basis of previous reports [6–10], which reported that the level of independence in dressing for stroke patients is associated with: balance function, motor and sensory function of affected side lower limb, the strength of the abdominal muscles, and unilateral spatial neglect (Fig 1). Therefore, we established a hypothesis describing which of these 5 functions has a direct effect on the level of independence in dressing in addition to the age and time post stroke. In addition, previous studies have reported that the balance function associates with motor function and sensory function of affected side lower limb [20], the strength of abdominal muscle [21] and knee extension of unaffected side [22], and unilateral spatial neglect [23,24], in stroke patients. Therefore, we set up a hypothesis in which these functions have direct effects on balance functions and indirect effects on dressing independence in addition to age and time post stroke.

As indicators of goodness of fit, we used the chi-squared test, Goodness Fit Index (GFI), Adjusted Goodness Fit Index (AGFI), Comparative Fit Index (CFI), and Root Mean Square Error of Approximation (RMSEA). The criteria for a good fit between the study data and the model were a GFI of at least 0.95, an AGFI of at least 0.90, a CFI of at least 0.95, and an RMSEA of less than 0.07 [25]. SPSS Amos 23.0 statistical software (IBM, Armonk, New York, USA) was used for all analyses. The level of significance was set at <5%.

## Results

**Fig 2:**
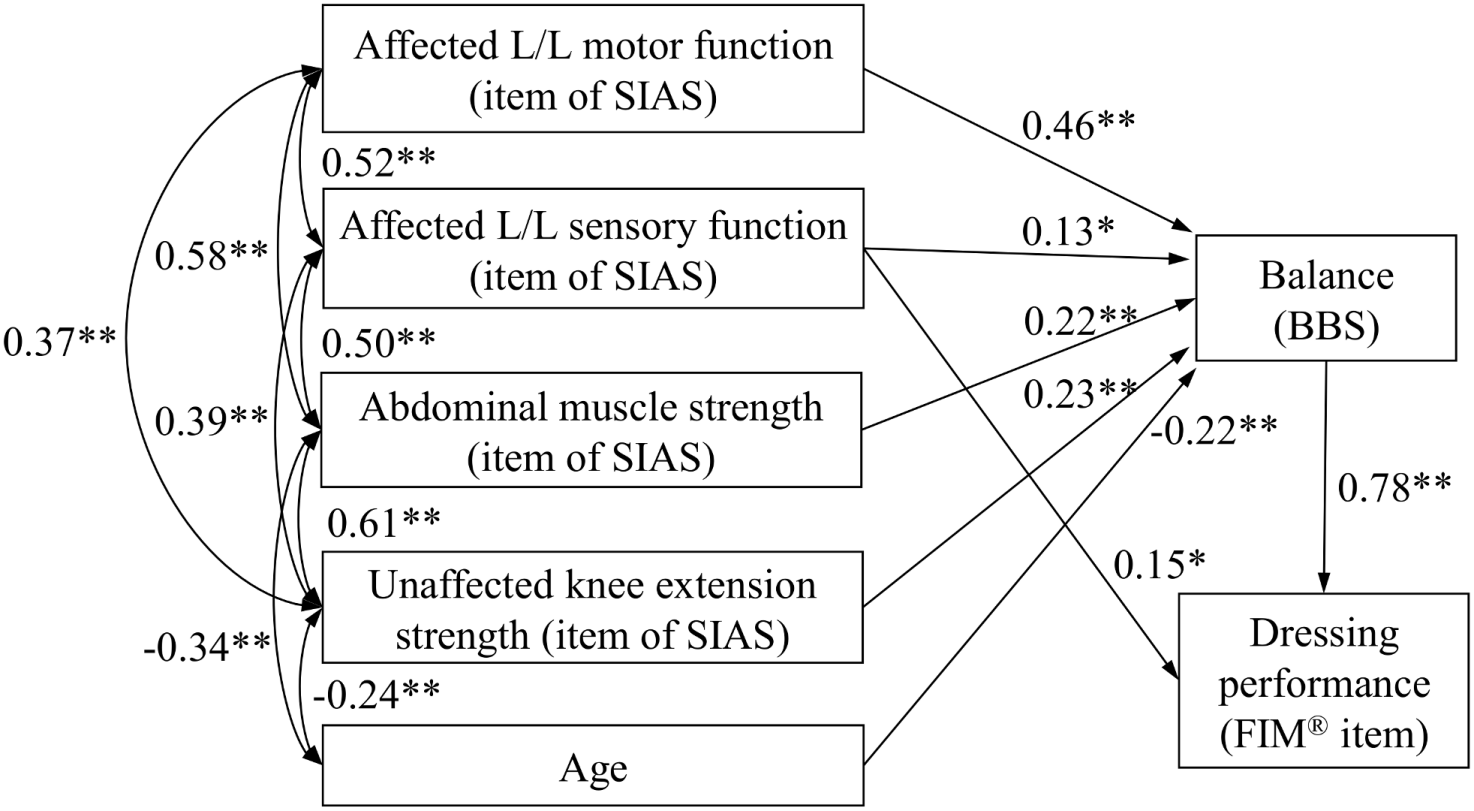
Modified model of the path diagram with the results of parameter estimates. A unidirectional arrow indicates a standardized path coefficient. A bidirectional arrow indicates a correlation coefficient. Abbreviations: L/L, Lower limb; SIAS, Stroke impairment assessment set; BBS, Berg balance scale. *P < 0.05, ** *P* < 0.01.

The assessment outcomes are presented in Table 1. The FIM^®^ instrument mean ± standard deviation score for dressing (1–7 points) was 4.3 ± 1.8 points. The indices of goodness of fit using the path model described in Fig 1 were χ^2^ = 0.326 (*P* = 0.568), GFI = 0.999, AGFI = 0.968, CFI = 1.000, RMSEA = 0.000, with all indices fulfilling the established criteria. However, non-significant path coefficients were also included in this model. Therefore, a modified path model that eliminated the non-significant paths was created (Fig 2). The indices of goodness of fit in the modified model presented in Fig 2 were χ^2^ = 4.760 (*P* = 0.575), GFI = 0.988, AGFI = 0.943, CFI = 1.000, and RMSEA = 0.000. All path coefficients were significant in the modified model, which accounted for 79.9% of the variance in FIM^®^ instrument score for dressing, and for 78.8% of the variance in BBS.

The standardized estimated direct effects of the BBS and the strength of abdominal muscles from SIAS on the FIM^®^ instrument score for dressing were 0.78 and 0.15, respectively. The standardized estimated direct effects of the motor and sensory functions of the affected side lower limb, strength of the abdominal muscles from SIAS, strength of knee extension on the unaffected side, and individual age on the BBS score were 0.46, 0.13, 0.22, 0.23, and −0.22, respectively. The standardized estimated total effects of the BBS score, motor and sensory functions of the affected side lower limb, strength of the abdominal muscles from SIAS, strength of knee extension on the unaffected side, and individual age on the FIM^®^ instrument score for dressing were 0.78, 0.36, 0.10, 0.32, 0.18, and −0.17, respectively.

## Discussion

This study demonstrated 2 major findings. First, the balance function and the strength of the abdominal muscles have direct effects on the level of independence in dressing in stroke patients. Second, the motor and sensory functions of the affected lower limb, the strength of the abdominal muscles and knee extension on the unaffected side, and age have indirect effects on dressing independence by mediating balance functions in stroke patients.

Previous studies have reported that the level of independence in dressing relates to the balance function [6,7], motor and sensory functions of the affected limb [8], strength of the abdominal muscles [9], and degree of visuospatial deficits [10]. Therefore, we established a hypothesis by which all these functions are related to the level of independence in dressing. From the initial model, we created a modified path model that eliminated the non-significant paths. This modified model was validated by the goodness of fit between the model and data, and all path coefficients were significant in the modified model. The model could account for more than 79.9% of the variance in the FIM^®^ instrument score for dressing.

In particular, the impact of balance function on dressing performance was very strong. This result is in agreement with a previous study that reported a close relationship between the level of independence in dressing and balance function [6,7]. Because dressing involves gross movement of the limbs and a shift in the center of gravity when sitting or standing, the level of independence in dressing can depend on balance function. In addition, the strength of the abdominal muscles contributes to trunk stability, and trunk stability is important for maintaining antigravity postures such as sitting and standing as well as smooth execution of limb movements [26–28]. The strength of the abdominal muscles may contribute to the level of independence in dressing by stabilizing the trunk. On the other hand, our study revealed that motor and sensory functions of the affected lower limb, strength of knee extension on the unaffected side, and age also contribute to the level of independence in dressing, but indirectly through their influence on balance function. Considering their impact on balance function, the strongest effects were from the motor function of the affected lower limb, followed in decreasing order by the strength of knee extension on the unaffected side, the strength of the abdominal muscles, and the sensory function of the affected lower limb.

These findings suggest that among stroke patients, dressing performance strongly depends on the balance function. Consequently, to improve dressing performance, training that focuses on improving balance function should be recommended. In our experience, a basic practice that improves dressing performance is repetitive dressing training because it facilitates the learning of compensatory movement techniques involving the unaffected upper limb and also improves balance functions. However, previous studies have shown that the amount of balance required for independent dressing with and without supervision or setup from caregivers was marked by BBS scores of 32 and 44 points, respectively [6]. The present findings indicate that high-level balancing function is required for dressing independence. Therefore, with a focus on improving balance function, repetitive training with dressing may not be sufficient, and it may also be necessary to add balance-specific exercises to achieve effective improvement in dressing performance.

In addition, our results suggested that the exercises that improve motor and sensory functions of the affected lower limb, strength of the abdominal muscles, and strength of knee extension on the unaffected side can improve the ability to balance, thus improving dressing performance. In particular, our results suggest that the motor function of the affected lower limb and the strength of the abdominal muscles are very important because they have large effects on the balance function. Previous studies have shown that trunk muscle strength has deteriorated in patients with stroke when compared with the strength in healthy age-matched subjects [29], and the greatest impairment is in forward flexion strength [29]. In addition, the strength of abdominal muscles or trunk flexion has been associated with dressing performance [9] and balance [21] in previous reports. Therefore, it is possible that the exercises focused on not only the lower limb motor function but also on strengthening the abdominal muscles will effectively improve individual balance function and dressing performance. In addition, both the present study and a previous report [20] have indicated that the sensory functions of the affected lower limb can impact balance function, although this effect was somewhat limited compared with impact of altered motor functions. It was recently reported that proprioception training alone or in combination with imagery exercises improved stroke patient perception of joint positioning and ability to balance [30]. For stroke patients with severe sensory disturbances, proprioception exercises may be an effective means of improving balance.

There are some limitations in this study. One of the limitations is that no psycho-social variables were addressed. In addition, a stratified analysis based on stroke severity and affected side was not performed. Moreover, the stroke patients in this study had a large range of demographics and stroke characteristics, and this study involved only a small sample size. Additional research is warranted to address these limitations and validate the study findings.

## Supporting Information

S1 Table Stroke Impairment Assessment Set’s scoring criteria (Excerpt of the item used by this research). (PDF)
