# Correction: Hierarchy of Dysfunction Related to Dressing Performance in Stroke Patients: A Path Analysis Study

**DOI:** 10.1371/journal.pone.0170519

**Published:** 2017-01-12

**Authors:** Takaaki Fujita, Hirofumi Nagayama, Atsushi Sato, Yuichi Yamamoto, Kazuhiro Yamane, Koji Otsuki, Kenji Tsuchiya, Fusae Tozato

Fig 2 is incorrect. The arrow from “Affected L/L sensory function (item of SIAS)” to “Dressing performance (FIM^®^ item)” is incorrect. The authors have provided a corrected version here.

**Fig 2:**
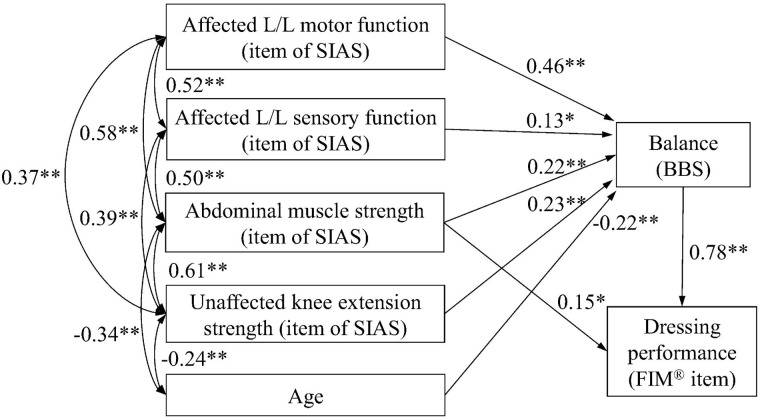
Modified model of the path diagram with the results of parameter estimates. A unidirectional arrow indicates a standardized path coefficient. A bidirectional arrow indicates a correlation coefficient. Abbreviations: L/L, Lower limb; SIAS, Stroke impairment assessment set; BBS, Berg balance scale. *P < 0.05, ** P < 0.01.
