# Supplementary material for: Hierarchy of Dysfunction Related to Dressing Performance in Stroke Patients: A Path Analysis Study
Source: PLoS One. 2016 Mar 8;11(3):e0151162. doi: 10.1371/journal.pone.0151162 (PMC4783045; doi:10.1371/journal.pone.0151162)
Supplement: S1 Table — (PDF) [file pone.0151162.s001.pdf]

S1 Table. Stroke Impairment Assessment Set's scoring criteria (Excerpt of the item used by this research)

MOTOR FUNCTION OF LOWER LIMB<br>- Hip flexion test<br>- Knee extension test<br>- Foot pat test<br>A score of 5: the patients can complete the task as smoothly as on the unaffected side.<br>A score of 3: the patient can complete the task with clumsiness.<br>A score of 0: no contraction of muscles<br>A score of 4,2,1: intervening levels of ability<br><br>SENSORY FUNCTION OF LOWER LIMB<br>- Light touch sensation<br>- Position test<br>A score of 3: normal<br>A score of 0: anesthesia<br>A score of 2,1: intervening levels of ability

ABDOMINAL MUSCLE STRENGTH

The patient rests in the 45 degree semireclining position in a wheel chair or high-back chair and is asked to raise the shoulders off the back of the chair and assume a sitting position.

A score of 3: the patient is able to sit up against considerable resistance

A score of 2: the patient can come to the sitting position despite pressure on the sternum by the examiner

A score of 1: the patient can sit up provided that there is no resistance to the movement

A score of 0: the patient is unable to sit up

QUADRICEPS MUSCLE STRENGTH OF UNAFFECTED SIDE

A score of 3: normal strength

A score of 2: minimal weakness

A score of 1: moderate weakness (grade 4 of the MMT score)

A score of 0: severe quadriceps weakness for the patient's age (around antigravity strength)

VISUOSPATIAL PERCEPTION

The patient is asked to touch the mid-portion of a tape held horizontally in front at a

distance of about 50cm. Two trials are allowed and the largest error is used for scoring.

A score of 3: deviation from the mid point by less than 2cm.

A score of 2: an error between 5 and 2cm

A score of 1: an error between 15 and 5 cm

A score of 0: more than a 15cm deviation from the central point

(Chino N, Sonoda S, Domen K, Saito E, Kimura A. Stroke impairment assessment set (SIAS) -a new evaluation instrument for stroke patients. Jpn J Rehabil Med 1994;31:119-25.)
